# Preferences for different insomnia treatment options in people with schizophrenia and related psychoses: a qualitative study

**DOI:** 10.3389/fpsyg.2015.00990

**Published:** 2015-07-14

**Authors:** Flavie Waters, Vivian W. Chiu, Aleksandar Janca, Amanda Atkinson, Melissa Ree

**Affiliations:** ^1^Clinical Research Centre, Graylands Hospital, North Metropolitan Area Health Service-Mental Health, PerthWA, Australia; ^2^School of Psychiatry and Clinical Neurosciences, University of Western Australia, PerthWA, Australia; ^3^Osborne Park Hospital, North Metropolitan Area Health Service-Mental Health, PerthWA, Australia; ^4^The Marian Center, PerthWA, Australia

**Keywords:** sleep disorders, sleep, insomnia, psychological treatment, CBT, melatonin, antipsychotics, neuroleptic

## Abstract

Symptoms of psychosis such as hallucinations and delusions can be intrusive and unwanted and often remain treatment-resistant. Due to recent progress in basic and clinical sciences, novel approaches such as sleep-based interventions are increasingly becoming offered to address the physical and mental health issues of people with severe mental illness. While the primary outcome is to improve sleep, studies have demonstrated that interventions that target symptoms of insomnia can also produce improvements in the severity of psychotic symptoms, quality of life, and functional outcomes. This study presents qualitative data on the attitudes and preferences of people with schizophrenia and schizo-affective disorders to three different types of therapies for insomnia (standard pharmacological, melatonin-based, and cognitive and/or behavior therapy). Interviews included discussions regarding the perceived advantages and limitations of different therapies, enablers to taking up the preferred option, as well as personal strategies that have helped respondents with sleep problems in the past. Results showed that, when given the choice, these individuals prefer psychological and behavioral-type therapy to other sleep interventions because of its potential to support and empower them in taking responsibility for their own recovery. Pharmacological therapies, by contrast, are viewed as useful in managing acute sleep problems, but only as a short-term solution. Overall, the findings underscore the need for patients’ active engagement when making decisions about treatment options.

## Introduction

The term ‘psychosis’ refers to a set of experiences which include hearing voices and seeing images that other people do not (‘hallucinations’) and unusual beliefs that others do not share (‘delusions’). These symptoms often attract a psychiatric diagnosis such as schizophrenia, schizoaffective disorder, bipolar disorder, or other psychotic disorders. Symptoms related to psychosis can be particularly distressing and burdensome, and can persist despite intensive and prolonged treatment, prompting the need to explore alternative interventions.

Sleep has been an area of increasing interest in the treatment of mental illness such as depression (Hickie et al., 2013) and more recently in the treatment of psychotic disorders (National Institute of Mental Health [NIMH], 2015). Insomnia is a particularly common condition with heterogeneous origin and which affects almost all individuals with psychosis at some point during their illness (Donlon and Blacker, 1975; Benca et al., 1992; Chemerinksi et al., 2002; Chouinard et al., 2004). Symptoms include highly irregular timing of sleep–wake patterns, difficulties getting to sleep or staying asleep, and being active at night and excessively tired during the day. Studies have demonstrated that these sleep problems are intrinsically linked to the cause and maintenance of psychotic symptoms (Zarcone and Benson, 1997; Yang and Winkelman, 2006; Waters et al., 2011) and to psychotic relapse (Heinrichs and Carpenter, 1985; Falloon, 1992). Individuals with psychosis and co-occurring sleep–wake problems are also more likely to have cognitive impairments, negative mood, reduced perceived ability to cope, daytime dysfunctions (Zarcone and Benson, 1997; Chemerinksi et al., 2002; Hofstetter et al., 2003; Chouinard et al., 2004) and an increased risk of suicide (Pompili et al., 2009).

In response to these findings, specific strategies which target poor sleep in psychosis are increasingly being developed with a view to improve sleep, but also to treat psychotic symptoms (e.g., Edinger et al., 2009; Myers et al., 2011; Wagley et al., 2013) and ameliorate clinical outcomes and functional disability (e.g., Suresh Kumar et al., 2007). The evidence-base, however, only so far includes small pilot studies, and there are no clear recommendations regarding the most effective treatment approach for insomnia in psychosis. In addition, questions remain regarding the extent to which these individuals are likely to take up sleep therapies.

Recovery-oriented practices advocate for individuals to be actively involved in decision-making about available intervention options, yet people living with schizophrenia and psychosis may not be given many choices or may find themselves unheard by mental health professionals in treatment planning and care (González-Torres et al., 2007). If interventions for insomnia are to be a viable treatment option, a key first stage must be to seek the personal perspectives of people with psychotic disorders regarding their preferences for different types of sleep interventions.

In this study, we sought to understand the preferences of a sample of individuals with psychosis regarding different types of therapies for insomnia. Qualitative interview methodology was used in the current study to identify preferred choices amongst different alternatives (‘testing alternatives’ analytic framework), and to identify issues of personal importance with regards to taking up these therapies (‘key concepts’ framework). Qualitative methods are often utilized to conduct in-depth inquiries into a specific issue and have considerable explanatory power when seeking to understand personal perspectives that cannot be comprehensively addressed using quantitative measures. New knowledge is generated through the analytical examination of narratives in response to semi-structured interviews. Typically, interviews and focus groups are conducted until the same themes keep re-emerging (a state of ‘saturation’). Thus, sample sizes in qualitative studies are typically much smaller than cross-sectional studies (and can be as small as *n* = 1). The lived experience of people with psychosis has previously been examined using such qualitative methodologies and studies show that these individuals are highly capable of engaging in this style of interviewing and providing informed opinions (Holmes et al., 1995).

This paper presents a report on the views and preferences of a sample of individuals with a diagnosis of schizophrenia and schizoaffective disorders regarding three different types of evidence-based therapies for insomnia: standard pharmacological, melatonin-based, and psychological and behavioral-type (talking) therapies. The interviews included discussions of (i) the perceived advantages and limitations of each therapy, (ii) preferred approaches, (iii) enablers to taking up the preferred therapy, and (iv) personal strategies that have helped these individuals with sleep problems in the past. Before the results are presented, an overview of the different treatments available, their evidence-base and their mechanisms of action, are presented.

## Insomnia Treatments Available for Psychosis

Different types of interventions are available to treat sleep dysfunction in psychosis. Some are based on techniques known to be effective in community samples and others are informed by an analysis of sleep–wake behaviors. The most commonly used interventions to regulate sleep–wake patterns and improve symptoms of insomnia include (1) standard pharmacotherapy, (2) melatonin, and (3) psychological and/or behavioral interventions. Other interventions exist (e.g., bright light therapy, stand-alone sleep restriction therapy) but insufficient evidence exists for their use in psychosis or they are not recommended due to a possible association with psychotic relapse (e.g., Sit et al., 2007; based on case study in bipolar disorder).

### Pharmacotherapy

A common clinical approach for managing insomnia in psychosis uses pharmacotherapy, with options including antipsychotics and hypnotics. The appeal of pharmacotherapy is its relatively rapid onset of action (Hickie et al., 2013) and sedatives and hypnotics are frequently prescribed for acute sleep problems (Falloon et al., 1996).

With regards to antipsychotics, the inhibitory effects of these drugs on dopamine (DA) transmission are thought to represent a likely mechanism for producing sedation. While antipsychotics are not recommended as a direct treatment for sleep disturbance in clinical guidelines, a common perception amongst mental health staff is that antipsychotics can satisfactory addresses both symptoms of psychosis and chronic insomnia. Indeed, many patients report immediate symptom relief, but empirical evidence regarding the sleep-promoting effects of antipsychotics remains mixed. For example, improvements in total sleep time, sleep efficiencies and stage 2 sleep have been reported (Monti and Monti, 2004) but sleep problems persist (e.g., Keshavan et al., 1996; Lieberman et al., 2005), with antipsychotic medication accounting for less than 10% of the total variance in sleep quality (Waters et al., 2012). Another factor of central importance includes the undesirable physical and somatic side effects of antipsychotic medications such as grogginess, next day tiredness, as well as difficulties getting to sleep or sleeping too much (e.g., Morrison et al., 2000; Ritsner et al., 2002).

### Melatonin

Melatonin (ingested as daily capsules) is commonly offered for the treatment of insomnia and other disturbances of the sleep–wake cycles in the general community. Melatonin is a natural hormone secreted by the pineal gland which acts to stabilize circadian rhythms. The sedative and chronobiologic effects of melatonin have led to its popular use as a treatment for insomnia, jet lag, circadian rhythm sleep disorders as well as in mood disorders. Melatonin produces fewer side-effects than antipsychotics, although it can be linked to headaches and drowsiness in higher dosages.

Given alterations in circadian chronobiologic rhythms of melatonin (phase advance, and low output) in individuals with schizophrenia-spectrum disorders (Van Cauter et al., 1991; Rao et al., 1994), melatonin appears to be a promising treatment option for insomnia. In support, studies provide some evidence for the beneficial effects of melatonin in this clinical group. Suresh Kumar et al. (2007), for example, conducted a randomized trial of melatonin versus placebo with 40 individuals with schizophrenia using flexible dose 3–12 mg of melatonin for 2 weeks as an adjunct to regular medication. Relative to placebo, melatonin had some benefits on night-time awakenings and sleep duration as assessed by questionnaires (small effect size), and was also linked to improvements in mood and daytime functioning. Using a smaller dose of 2 mg of melatonin and a sample of 19 individuals with schizophrenia in a randomized, double-blind, and cross-over design, Shamir et al. (2000) demonstrated that melatonin treatment for 3 weeks was linked to improved sleep efficiency and sleep duration, as well as reduced duration to sleep onset, although these improvements were mostly observed in people whose sleep quality was low at baseline.

### Psychological and Behavioral Interventions (Including CBT)

Neurobiological changes can explain many of the sleep problems in psychosis (e.g., Van Cauter et al., 1991; Rao et al., 1994), although sleep habits and psychological problems also contribute significantly to the onset and maintenance of insomnia. For instance, precipitating factors for insomnia often include stressful life events, and there is a wealth of evidence demonstrating that psychological issues such as worry and rumination, and cognitive factors such as dysfunctional beliefs and thought control strategies, perpetuate symptoms of insomnia in the general community (Harvey, 2002). Therefore, management strategies which target psychological, cognitive, and behavioral factors that prevent sufferers from re-establishing a normal sleep pattern have recently been the subject of much attention.

Components of cognitive interventions target cognitive hyperarousal, maladaptive thought control strategies, dysfunctional beliefs and attitudes such as the perceived need to control sleep, and relaxation elements. By contrast, behavioral interventions focus on the formation of good sleep habits (a regular wake time, not clock watching, managing caffeine, and alcohol intake), breaking negative associations of the bed as a place of frustration, and awareness of sleep-promoting activities such as regular exercise. The benefits of such approaches to insomnia in the general population are well documented (e.g., Harvey et al., 2014).

Some therapeutic approaches include behavioral interventions alone (e.g., sleep restrictions), although studies in the (non-psychiatric) community show that therapies that address both behavioral and cognitive elements (‘Cognitive–Behavioral Therapy for Insomnia, CBT-I’) have the most potential in producing reliable and durable changes (Spielman et al., 1987; Perlis et al., 2005; Schmidt et al., 2011).

Individuals with psychosis often have a sedentary lifestyle and a lack of daytime structure (Hofstetter et al., 2003), suggesting that behavioral approaches promoting good sleep, regular sleep routine, and contributors to daytime sleepiness can therefore increase awareness about the need for behavioral changes. We recently demonstrated evidence of knowledge gaps about sleep hygiene in inpatients with psychosis, specifically regarding the importance of regular sleep–wake timings and the wake-promoting effects of nicotine (Chiu et al., 2015a, in 55 inpatients with psychotic disorders). In addition, these psychiatric individuals demonstrated levels of thought control strategies (rumination and aggressive suppression) and dysfunctional beliefs about sleep (preoccupation about the consequence of poor sleep) that were similar to non-psychiatric controls insomnia, suggesting that interventions combining cognitive and behavioral strategies (such as CBT-I) are likely to be most efficacious for treating insomnia in psychosis.

While CBT-I is recommended by the American Academy of Sleep Medicine as a first-line treatment for insomnia (Espie, 2009), few studies has been published in people with psychosis. Myers et al. (2011) provided four individual CBT-I sessions to 15 patients with persisting persecutory delusions and insomnia. It was reported that participants reported significant improvements in self-reported sleep quality as well as reductions in delusions and psychological distress at post-intervention and one month follow-up, providing ‘proof of concept’ for CBT-I in psychosis. Other studies have reported on the effectiveness of cognitive and behavioral interventions, although these other studies included mixed diagnostic groups with primarily affective or anxiety disorders (Edinger et al., 2009; Wagley et al., 2013) or abbreviated treatment components (one session Lyne et al., 2011; two sessions Wagley et al., 2013).

Overall, a number of interventions may be used to improve sleep and symptoms for people with schizophrenia and related psychoses. The current paper now presents a study of their preferences regarding these interventions.

## Qualitative Study into the Views and Preferences of Respondents Regarding Three Different Types of Insomnia Treatments

In this section, the views and preferences of a sample of individuals with schizophrenia and related psychoses with regards different types of evidence-based therapies for insomnia are presented. The interviews included discussions of (i) the perceived advantages and limitations of these therapies, (ii) preferred approaches, (iii) enablers to taking up the preferred therapy, and (iv) personal strategies that have helped them with sleep problems in the past.

### Participants

Participants were recruited from Graylands Psychiatric Hospital (inpatient wards) and a local mental health drop-in centre (The Lorikeet Centre). Recruitment occurred using advertising and a snow-ball technique. A total of 14 respondents completed this study (aged 28–64; 50% males). In keeping with previous research conducted with this population (Carey et al., 1999), the aim was for focus group with sizes of three to four people. Three focus groups (comprising up to four people), and six individual interviews for individuals who preferred not to engage in groups were conducted between November 2013 and January 2014.

All individuals had been given a diagnosis of schizophrenia or schizoaffective disorder by a mental health physician, and all participants had substantial past or current sleep problems accompanied with daytime dysfunction. Length of illness since first hospitalization ranged from three to 33 years (mean = 16 years). All participants were taking one or more antipsychotic drug; three were also taking benzodiazepines, and five antidepressants. All had a prior experience of sedative and/or benzodiazepine. One participant had received sleep hygiene information in the past (delivered informally by a mental health professional). Two had tried melatonin, and one (ID4.1) was still taking melatonin, but none had received CBT-I. Ethical approval was granted by the University of Western Australia and North Metropolitan Health Service – Mental Health Human Research Ethics Committees.

### Methods

Focus groups followed standard procedures (Morgan et al., 1998). Focus groups and individual interviews were conducted by psychologist VC at the drop-in centre or at Graylands Hospital. Oversight was provided by a senior team member (FW or MR) to ensure that the procedures were applied consistently, that participants had sufficient time and opportunities to give their views and that in-depth discussions were pursued. Groups began with an introduction, including conditions of confidentiality and respect for one another, followed by the methods and rationale of the project. Topics were addressed in a flexible way using a semi-structured interview schedule developed by the investigators. In Part 1, personal experiences of sleep problems, help seeking behavior, therapies that had been tried (including standard pharmacologic), and personal strategies that helped in the past were addressed. In Part 2, a handout was provided with information about melatonin and cognitive/behavior therapies drawn from the literature and consumer medicine information. No handouts were provided for pharmacological therapies since all participants had prior experience of antipsychotics and sedatives. Discussions focused on the perceived benefits of different therapies (‘what do you like most?’) and limitations (‘what do you like least?’), preferred approaches for addressing sleep problems in the short term and in the longer term, and what would help them to take up the preferred interventions. Prompts were used to aid discussion and encourage participants to follow on from previous comments. After the consent and introduction, discussions lasted 45–60 min.

#### Analysis of Transcripts

Sessions were recorded on audio tape, and then transcribed using Krueger and Casey’s (2000) approach to sorting transcript data. Two researchers (VC and FW) made themselves familiar with the content of transcripts. VC then coded the transcripts, and sorted the coded data into categories and subcategories in excel document spreadsheets (descriptions of sleep problems, impact, coping, treatment types, enablers, personal strategies) which were verified by the other researchers (FW and MR). Next, the research team analyzed the spreadsheets independently and then met to compare and discuss the coded data and whether some categories should be combined or separated until a consensus was reached. No difficulties in coding were met and interrater agreement was high with no disagreements arising on the main findings. The joint discussions also allowed team members to reflect on their views of the research, and the representation of multiple specialists in the team allowed an analysis and interpretation of the data without any systematic biases. Credibility checks were conducted with additional consultation with a study participant and with the input of person with a lived experience.

## Results of the Qualitative Study Interviews

### Attitudes Toward, and Preferences for, Different Types of Insomnia Therapies

Using the systematic analysis described above, reports were pooled separately for the different interventions. Overall, the respondents had firm opinions about the different sleep treatments (see **Table 1** for summary).

**Table 1:** Perceived benefits and limitations of different types of sleep interventions.

	Pharmacotherapy	Melatonin	Cognitive and/or behavioral therapies
Acceptability	22.5%	57.2%	71.5%
**Benefits**			
Improves sleep routine		√	√
Long term solution to sleep problems			√
Drug free			√
Helps with worries, stress, racing thoughts			√
Human contact in therapy			√
Regaining control			√
Learn something new			√
**Limitations**			
Limited perceived efficacy for treating sleep			√
Interaction between drugs	√	√	
Tiredness as a side-effect	√	√	
Poor concentration as side-effect	√		
Lack of control over the process	√	√	
Lack of motivation as possible barrier			√

**Table 1** shows seven elements which participants identified as valuable in a sleep therapy and which address not only sleep issues (routine, long-term solution), but also physical health (drug-free), psychological health (worry, stress, intrusive thoughts, and social contact) and principles sustaining recovery (regaining control, and learning something new). Cognitive and behavioral therapies met all seven criteria and was rated as acceptable by 71% of participants, melatonin met one criterion (acceptable in 57.2% of the sample), and pharmacotherapy none (acceptable by only 22.5% of participants).

Six elements were then identified as unwanted in therapy: lack of confidence in its evidence base; risks associated with polypharmacy; side effects from medications (cognitive and functional effects); and lack of choice and control over the process. Lack of motivation was also perceived as a barrier to engaging in therapy. Pharmacotherapy met 4/6 (66%) of these unwanted elements, melatonin 3/6 (50%), and cognitive and behavioral therapies 2/6 (33%).

#### Pharmacotherapy

Respondents reported mixed feelings about their medications, and had both positive and negative opinions about pharmacological approaches to sleep treatment. Six respondents (42.8%) felt that medications had helped with their sleep problems:

(ID7.1) Well I went to the psychiatrist and she prescribed me antipsychotics and I went to sleep after that.

(ID4.1) I’d like to have been on something else, like temaz just for a little while to cover the time so I can have a decent night’s sleep.

A majority (*n* = 11, 78.5%) however, did not view pharmacotherapy as an acceptable intervention. Reasons included unwanted side effects and possible interactions between drugs (*n* = 7, 50%):

(ID8.2) I was just about to say, sometimes it takes about 11 or 12 hours I’ve noticed with some medications to wear off enough to be alert you know what I mean?

Other reasons were that the medications didn’t always help with sleep problems (42.8%), or that their physicians were often changing the type, dosage or scheduling of medications, which impacted on their sleep:

(ID6.1) I couldn’t sleep for weeks … the doctor in her wisdom decided to change my medication; They’re always juggling with it in the clinics, and I’ve had to put my foot down – no more juggling unless there’s a really good reason for it, I don’t wanna be a guinea pig.

#### Melatonin

Respondents’ views of melatonin were largely favorable. Six respondents (42.8%) were drawn by its novelty and had not heard of other people using it:

(ID1.2) Probably it’s just something new. You wanna try something new, see if it will help me go to sleep.

More than a quarter of the sample (*n* = 4; 28.5%) viewed melatonin as a more natural and safer option than pharmacotherapy:

(ID9.1) If you’re giving melatonin works better than a sleeping tablet, it’d be a more natural option.

(ID6.1) Well I know that melatonin’s natural and it is something natural.

While melatonin was perceived to be ‘more natural’ than pharmacotherapy, it was still viewed by many as another drug option, and thus potentially harmful. Six participants (42.8%) did not want to take more medications than they needed to:

(ID2.1) Aw the thing is I don’t…I take plenty of medications and I wouldn’t want to take any more than I have to…

(ID3.2) Yeah that’s the thing, like you know, for years with me like I’d take tablets, a side effect pill to counteract that, and injections too… and now I’m down to just a couple of tablets at night and I don’t want to go back to scoffing down pills and getting jabs you know

A sizeable minority also worried about possible side effects (*n* = 6, 42.8%), particularly relating to headaches and tiredness, and the interaction melatonin might have with other drugs:

(ID4.1) I’d rather not be on melatonin if it can cause headaches or drowsiness.

(ID1.3) What if it clashed with the other medication that we’re taking?

#### Cognitive and/or Behavioral Interventions

Discussion of non-pharmacologic interventions brought up a greater number of positive than negative points, and a quarter of respondents did not raise any negatives views toward cognitive and behavioral interventions (29%).

Half of all respondents (50%) agreed that a benefit of such treatment was that it was a drug-free option:

(ID3.1) Because it doesn’t include much of the things with medications. I get some pretty bad things with side-effects.

(ID5.1) Learn to know yourself better, because spend time to think about what affects you or not, something good for you, natural way.

However, a quarter of respondents (*n* = 4; 28.5%) were skeptical regarding whether it could offer something new that they had not tried before, or stated that ‘talking’ had limited efficacy on sleep problems:

(ID5.2) yeah cause I’ve tried a lot of like CBT kind of stuff, and what else… yeah I’ve tried heaps of stuff. I got into Buddhism like full on, and I was meditating every day, and guided visualizations… all that sort of stuff, and it doesn’t seem to do anything.

Being actively engaged in the treatment process was a benefit of cognitive/behavior therapy for 50% of respondents, who felt that being involved in the treatment process would give them a sense of control and autonomy:

(ID1.3) Like working at it and doing.. you know…what we’ve been told…working at it. Like trying to help yourself.

(ID3.2) But if you said, like it’s a test and you make that decision and that’s what you want to do, then that’s your decision, your control…so it’s a bit of a plus there. Yeah I think that’s pretty much a human element isn’t it?

The majority of respondents (57.1%, *n* = 8) understood that the aims were to establish a good sleep routine and to promote long-lasting changes in sleep behavior.

(ID6.1) Yeah. I’d give it all I had you know, because I’ve grown to understand what it…I’m looking to break the bad sleeping habits really. It probably works better in the long term, I think, because what you learn stays with you… well that might be my experience.

Sleep problems in psychosis are often related to worries, stress, and anxiety. Respondents stated they would value therapist advice on how to deal with stress and racing thoughts (28.5%, *n* = 4). Learning to relax was also perceived to be a valuable skill by four participants (28.5%):

(ID1.2) They’ve got a progressive one which relaxes your muscles from there down to your feet which is very, very good

Conversely, two participants (14.3%) worried those psychological therapies for sleep may bring up traumatic issues;

(ID1.2) Well it might be a bit unsettling too…Well it might stir up too much stuff that you can’t deal with. Yeah, there’s people who may be sensitive to it.

Finally, face to face interaction, and the sharing of personal experiences, was seen to be of therapeutic benefit for four respondents (28.5%):

(ID8.2) I used to ring helplines and things like that. I’ve had friends too who have rung me at odd times of the night. I think sometimes all you need is some confirmation you’re human, you’re alive.

(ID4.1) About life issues, about problems, you can keep people calm, not be frightened of sharing your thoughts, to have understanding of one another.

Challenges for 42.8% of participants (*n* = 6) included the discipline, time and hard work that is required for the psychological therapy. The motivation needed to repeatedly attend the sessions and actively engage in treatment was also voiced as a limitation by five (35.7%) of the respondents:

(ID5.2) You see my biggest problem is my motivation, I’m not very motivated. I’m told that that’s part of something to do with the frontal lobe or something like that where it affects my motivation, but I’m not sure if that’s sleep-related or not

### Participants’ Views Regarding How to Best Support their Sleep

#### Preferred Approach

Participants were asked to reflect back on their journey with mental illness and to describe the type of insomnia treatment they would chose if they had been given the choice.

One dominant opinion (57.1%, *n* = 8) was that one approach might not fit everyone, all of the time. Specifically, a treatment might work for one person but not another, and also that some interventions work better at some stages of the illness or recovery than others. Particularly, during the acute phase of the illness, pharmacological approaches might work best:

(ID6.1) In those days, I was just hopelessly entwined in my illness and I thought I was alright, but looking back like…

(ID5.1) Sometimes I can’t work by myself, don’t know why and only the medicine can keep me in routine.

Respondents also suggested that psychological and behavioral interventions should be the first line treatment, with a focus on sleep education, relaxation techniques and having someone to speak to, and with pharmacological options available if cognitive and behavioral sessions do not seem to be improving their sleep.

(ID2.1) When that person starts getting aggravated and saying “this isn’t working” And then they start getting aggro “I can’t sleep, f^∗∗^ off, and I can’t do this anymore”, that’s maybe when the medication should come in. But it should be kept at a bare minimum, you know, only like you know if they use it once every 5 days that would be really good you know.

#### Enablers of Psychological and Behavioral Therapies for Sleep Problems

With cognitive and behavioral therapies as the preferred option, we asked participants about factors that might enable and support them in taking up such therapies. **Table 2** shows that participants provided a wide range of solutions.

**Table 2:** Enablers to psychological sleep therapies.

Main themes	Enablers
(1) Information that is relevant to people with psychosis	• *Knowing that it would improve sleep* •*I think hearing about the success rates. Hearing… the outcomes would make people wanna go more* •*Make the sessions and the instruction content easy to understand* •*I think it should be worded in such a way that I would understand*
(2) Sleeping better is linked to feeling better	• *The biggest motivation is the intrinsic improvement* •*Yeah, if you see improvement it will help you stick to it* •*It’s good to have things to look forward to…you know, like once you get your rest either way, you’ll start feeling better, you’ll think better, you’ll have more energy, and yeah umm...*
(3) Empowerment and teaching others	•*If one person’s motivated, maybe someone else will get motivated and, if you’re lucky, half the people get into it* •*Just adding to my general knowledge base you know, and being able to help other people* •*Being able to talk about the experience of doing it and what worked for me, and maybe consider if they make the judgment of what might work for them*
(4) Overcoming personal challenges	•*Learn how to let go so that I can sleep; you know, let go mentally and physically so I can sleep, that would be the overriding thing.* •*Yeah well probably self-talk would come into it, because I know if I can talk myself into getting out of bed early, it’s a big hurdle to overcome – that’s one of my bad habits…* •*Like take your medication at a set time, do some sort of ritual* •*There are lots of stumbling blocks, but I’m just learning how to use my brain now. I have... was in and out of hospital for at least 5 years with no drugs, no crime… and uh that helped a lot with everything*
(5) Practical issues	•*Well little things like catching a taxi motivated me to come today. Um so just, just um... just making things easier*

Solutions were broken down into five main themes. The first theme relates to access to information in a language that is easy to understand, and which addresses sleep issues that are directly relevant for people with symptoms of psychosis. Therefore, studies which seek to understand the perspective and personal experiences of people with lived experience, in their own words, are better able to identify gaps in knowledge and define clinical targets. For instance, our recent work with groups of people with psychosis suggests that night-time rumination, worries, hallucinated voices, external locus of control (Chiu et al., 2015a), as well as frequent nightmares, night terrors (Chiu et al., 2015b) are key experiences which prevent good sleep from happening. In other words, while better sleep is an objective, targets for treatment should first address factors that get in the way of achieving this objective.

Themes 2 and 3 address some of the benefits of therapies from a personal perspective, and using the group format as a productive conduit. First, sleep improvements are linked to broader psychological improvements which include feeling better, reduced tiredness and increased energy, all of which provide motivation to persevere with the therapy. Second, group interventions are perceived as advantageous by providing opportunities to contribute and learn as a valued member of their community. The opportunity to talk to and learn from others was important for many participants, who reflected that each individual has a personal and unique journey which can be meaningfully shared with others facing similar issues.

Theme 4 relates to overcoming personal challenges and the hard work necessary to make changes in sleep habits and cognitions that support good sleep. The ideas raised were drawn from each individual’s experience in finding their own personal strengths – some learnt to ‘let go,’ others to ‘self-talk,’ or ‘conduct rituals.’ These self-reflections make psychological therapies particularly suitable for addressing sleep problems (as opposed to the use of melatonin or pharmacological therapies alone) given that they provide a space for the person to find their strengths and personal strategies as guided by an experienced therapist.

Finally, theme 5 relate to practical issues about attending the sessions. One participant raised the need for transport assistance to attend the session. Financial hardships experienced by people with psychosis on a pension are a common problem, and can get in the way of these individuals receiving the right treatment and at the right time.

#### Strategies that have Helped with Sleep Problems or for Going Back to Bed

Finally, participants’ strategies that had been found to support their sleep were solicited (**Table 3**). Sleep tips are now widely available on the internet and popular media (for example the Sleephealthfoundation.org.au) and from general and sleep practitioners. Yet few are written for people with psychotic disorders, and by people who themselves experience psychosis. Although the current participant group had not received any coaching or formal sleep education, their strategies closely followed those recommended for community populations such as the importance of winding down, relaxation, and regular bedtimes.

**Table 3:** Personal strategies that have helped with sleep problems and for getting back to sleep.

**Routine** • A routine before bed like a ritual that is repeated every night • Drinking milk before going to bed • Reading a book • Take a bath **Sleep hygiene** • More exercise during the day • Quitting smoking and drugs **Relaxation** • Relaxation methods like meditation • Relaxing music **Addressing negative thoughts** • Write in a book what you are thinking before you go to bed • Do some art before going to bed to resolve an issue • Doing an activity that breaks the train of thoughts that is going through your mind which is stopping you getting to sleep **Feeling supported emotionally** • Ring the Samaritans so that I can tell someone what is on my mind, and I feel validated • Talking to someone

Negative thoughts at bedtime were commonly brought up as needing coping strategies. Worries and intrusive and ruminative thoughts are a common feature of psychotic disorders (Morrison and Baker, 2000) and are particularly elevated in those with symptoms of insomnia (Chiu et al., 2015a). It is thus particularly important that these individuals find strategies that address these psychological issues. Since CBT-I is the only type of therapy which specifically addresses negative thoughts at bedtime, this particular sleep intervention appears particularly well suited to address the needs of this population.

Finally, social and psychological support (‘feeling supported’) is often taken for granted by people in the community. One participant suggested ringing helplines as a way to meeting one’s psychological needs and reassurance, and achieving better sleep.

## Summary and Discussion

This paper examined the views and attitudes of people with psychosis about different treatments for insomnia. This study demonstrated, yet again, that people with severe and persistent mental illness are perceptive and insightful and must to be consulted about their treatment and care.

Respondents expressed mixed feelings about pharmacotherapy. Many felt that antipsychotic and sedative medications had benefited their sleep, although concerns remained about side effects, particularly next-day drowsiness, poor attention, and concentration. This supports recent findings reporting on the side effects of antipsychotics on concentration, memory, tiredness, and restlessness (Morrison et al., 2000) and of the distress caused by these reactions (Finn et al., 1990).

A majority of participants responded positively to melatonin as a sleep treatment option, and a majority said they might try it if given the opportunity. Its unfamiliarity, however, led to concerns about how it might interact with their other medications and other side effects.

In the decisional balance, psychological and behavioral-type therapies were perceived as more attractive and with the potential to have greater sleep, psychological, physical, and recovery benefits than any other type of intervention, and the majority of respondents favored this approach over the others. The dominant view was that such therapies have the potential to support and empower these individuals to take responsibility for their own lives and their recovery. Participants noted that the learning process regarding how to manage sleep, and how to cope with sleep problems, provides the only approach to a long-term solution to their sleep problems. This acknowledges that these individuals are experts in their own lives, and that they should be given ownership and responsibility for their choices with appropriate support.

While some respondents were skeptical regarding the potential of such therapies to help with their sleep, many were drawn to psychological support as a way of addressing stress, worries, and racing thoughts. Ambivalence regarding the value of CBT by clients with psychosis has been described previously. In Messari and Hallam (2003), clients expressed skepticism regarding the benefits of the therapies for improving symptoms but, at the same time, they valued the educational components of the program and their relationship with the therapist. This suggests that doubts should not be perceived as a barrier for engaging in treatment as multiple benefits might arise, including psychological improvements with downstream effects on sleep (Franzen and Buysse, 2008). In further support for ‘talking’ therapies, opportunities for human contact and emotional support were also high on the list of enablers for taking up therapy. It is often forgotten that many people with psychosis live alone and experience substantial loneliness. Two participants in this study, for instance, reported telephoning helplines in the middle of the night to achieve this basic human need. Psychological therapies are therefore highly suited to address these concerns which can act as a barrier to good sleep.

Lack of motivation was recognized as a possible barrier to engaging in psychological and behavioral therapies. Lack of motivation is a well-known symptom of psychosis (e.g., Bentall et al., 2010) which likely impacts on cognition and take-up of therapies (Gard et al., 2009). This suggests that therapies that include motivational methods to engage clients maintain treatment adherence and maximize outcomes are most likely to succeed (Gard et al., 2009). Interestingly, the current participants recognized that personal efforts and hard work are instrumental in regaining a sense of control, autonomy, and independence, placing value in intrinsic motivation with the identification of personal goals that have real-world relevance and which underlie recovery principles.

One possible motivation strategy makes use of group therapy with its opportunity for sharing lessons from one’s journey and to learn from each other. In support, in this study, the group format as a methodological approach led to the exploration of a broader range of issues relative to single interviews. Of note, however, a subgroup of participants chose to have individual interviews rather than a group session. While no key differences in sleep themes emerged, personal contents were more frequently brought up, with participants reporting a greater number of adverse experiences (effects of trauma, grief, and loss on sleep, and detailed descriptions of nightmares). It is possible that these experiences were also common in the other participants given that trauma is a common feature of psychosis (Tarrier et al., 2007), but that the group format was not conducive of such personal discussions. The current data does not provide answers to this question, but our findings suggest that the opportunity to have choices is important and that group therapy may not suit everyone.

When asked their opinion regarding which treatment might work best, participants explained that everyone is different, and that people need different things at different times. Cognitive and behavioral approaches were perceived as more likely to yield benefits in the longer term, but they may not suit all individuals especially if the person is experiencing lack of insight, uncontrollable tiredness, or an acute episode of illness. Eight participants thought that concurrent pharmacological and psychological/behavioral interventions might be the optimal approach during an episode of acute illness.

With regards limitations, the objective of the study was to sample the views of people with psychosis in different environments, and participants included both psychiatric inpatients and individuals in remission from psychotic disorders. One consequence, therefore, is a small sample size in each of the two groups, although this approach may arguably provide greater methodological triangulation. The primary difference between inpatients and outpatients was in the discussions by inpatients about their sleep experiences in the hospital environment (e.g., help from nurses, sleeping walking amongst patients in the wards). Another limitation is that individuals volunteered to take part in this study possibly introducing a participant bias. Finally, no participants had received CBT-I but all had used pharmacological approaches and two had tried melatonin. Participants had, therefore, informed views about antipsychotic medications and sedatives, and were more likely to be aware of their side effects. By contrast, participants made ‘educated guesses’ about the other treatments which may not reflect the views of a personal experience of these interventions.

## Conclusion

In light of evidence showing that poor sleep is intrinsically linked to symptoms of psychosis and that it may act as a barrier to recovery, it is becoming a clinical priority to fully address sleep problems in people with psychotic disorders. In this study, psychological and behavioral-type interventions and melatonin were viewed as valid adjuncts to pharmacotherapy and there was a strong desire for participants to make their own choices regarding the type, and timing, of their therapies. This suggests that psychological and behavioral treatments, including CBT-I, should be more systematically offered to people with psychosis with co-occurring insomnia. In services without clinical expertise in CBT-I, allied health professionals should offer behavioral interventions and education with an emphasis on good sleep habits (a regular wake time, not clock watching, managing caffeine and alcohol intake), awareness of sleep-promoting activities such as regular exercise, association of the bedroom for sleep only (removing TV, phones, etc, and not sleeping in living areas), as well as relaxation and stress-management techniques (Morin et al., 2006).

Overall, this study showed that clients seeking clinical care for sleep problems must be consulted about their preferences and views, and that engagement with others who share similar experiences and an understanding of advantages and limitation of different interventions may be key elements to successful change.

## Conflict of Interest Statement

The authors declare that the research was conducted in the absence of any commercial or financial relationships that could be construed as a potential conflict of interest.
